# Drug repositioning based on weighted local information augmented graph neural network

**DOI:** 10.1093/bib/bbad431

**Published:** 2023-11-28

**Authors:** Yajie Meng, Yi Wang, Junlin Xu, Changcheng Lu, Xianfang Tang, Tao Peng, Bengong Zhang, Geng Tian, Jialiang Yang

**Affiliations:** Center of Applied Mathematics & Interdisciplinary Science, School of Mathematical & Physical Sciences, Wuhan Textile University, No. 1, Yangguang Avenue, Jiangxia District, Wuhan City, Hubei Province 430200, China; Center of Applied Mathematics & Interdisciplinary Science, School of Mathematical & Physical Sciences, Wuhan Textile University, No. 1, Yangguang Avenue, Jiangxia District, Wuhan City, Hubei Province 430200, China; College of Computer Science and Electronic Engineering, Hunan University, Lushan Road (S), Yuelu District, Changsha, Hunan Province 410082, China; College of Computer Science and Electronic Engineering, Hunan University, Lushan Road (S), Yuelu District, Changsha, Hunan Province 410082, China; Center of Applied Mathematics & Interdisciplinary Science, School of Mathematical & Physical Sciences, Wuhan Textile University, No. 1, Yangguang Avenue, Jiangxia District, Wuhan City, Hubei Province 430200, China; Center of Applied Mathematics & Interdisciplinary Science, School of Mathematical & Physical Sciences, Wuhan Textile University, No. 1, Yangguang Avenue, Jiangxia District, Wuhan City, Hubei Province 430200, China; Center of Applied Mathematics & Interdisciplinary Science, School of Mathematical & Physical Sciences, Wuhan Textile University, No. 1, Yangguang Avenue, Jiangxia District, Wuhan City, Hubei Province 430200, China; Geneis Beijing Co., Ltd, No. 31, New North Road, Laiguanying, Chaoyang District, Beijing 100102, China; Geneis Beijing Co., Ltd, No. 31, New North Road, Laiguanying, Chaoyang District, Beijing 100102, China

**Keywords:** drug–disease association, drug repositioning, graph neural network, graph attention mechanism, local information augmentation

## Abstract

Drug repositioning, the strategy of redirecting existing drugs to new therapeutic purposes, is pivotal in accelerating drug discovery. While many studies have engaged in modeling complex drug–disease associations, they often overlook the relevance between different node embeddings. Consequently, we propose a novel weighted local information augmented graph neural network model, termed DRAGNN, for drug repositioning. Specifically, DRAGNN firstly incorporates a graph attention mechanism to dynamically allocate attention coefficients to drug and disease heterogeneous nodes, enhancing the effectiveness of target node information collection. To prevent excessive embedding of information in a limited vector space, we omit self-node information aggregation, thereby emphasizing valuable heterogeneous and homogeneous information. Additionally, average pooling in neighbor information aggregation is introduced to enhance local information while maintaining simplicity. A multi-layer perceptron is then employed to generate the final association predictions. The model’s effectiveness for drug repositioning is supported by a 10-times 10-fold cross-validation on three benchmark datasets. Further validation is provided through analysis of the predicted associations using multiple authoritative data sources, molecular docking experiments and drug–disease network analysis, laying a solid foundation for future drug discovery.

## INTRODUCTION

The development of new drugs remains a significant issue and challenge in the advancement of the biomedical field [1]. Over the past few decades, pharmaceutical research and development techniques have rapidly evolved alongside genomics, proteomics, life sciences and technological advancements [2]. The process of drug development, spanning from initial discovery to final market approval, typically spans around 10 years [3]. However, the majority of experimental drugs fail to progress beyond phase I clinical trials due to unforeseen adverse reactions caused by these novel medications [4]. According to the Tufts Center for Drug Development Research, the cost of developing a new drug amounts to $2.6 billion for pharmaceutical companies. This substantial expense is primarily attributed to ineffective compound selection during development and the challenge of identifying adverse effects and efficacy at an early stage in the process [5]. Consequently, there is an urgent need for effective strategies to enhance the efficiency of drug research and development, ultimately resulting in significant reductions in the research and development cycle and minimizing excessive investment in human resources, materials and finances. While wet experimental techniques can verify drug–disease interactions, they are characterized by their labor-intensive and time-consuming nature [6]. Computational methods have been extensively employed in bioinformatics and cheminformatics research for almost three decades [1]. As a result, computational drug repositioning methods have emerged as crucial approaches to expedite drug discovery.

Matrix factorization is a widely employed computational method in drug repositioning. It involves decomposing the known drug–disease interaction matrix into two low-rank matrices, representing drug features and disease features, respectively. By calculating the interaction probability scores of drugs and diseases based on their respective feature spaces, the relationship between them can be predicted. The matrix completion method assumes that the known drug–disease interaction matrix is incomplete, implying the presence of unknown interaction relationships. Thus, the objective of matrix completion is to predict and fill in the missing interactions using the available interaction information. Matrix factorization and completion algorithms have found successful applications in various domains of bioinformatics research. For instance, they have been utilized to predict microRNA–disease interactions [7], infer potential drug–virus associations [8, 9] and address the dropout issue in single-cell RNA sequencing through imputation modeling [10]. Many previous studies have demonstrated the feasibility of applying matrix factorization and completion methods to drug repositioning. For instance, Ai *et al*. proposed a low-rank matrix factorization algorithm that incorporates multi-graph regularization. This method effectively combines multiple similarity matrices through graph regularization to create smoother representations of disease and drug samples in the manifold space [11]. Mongia *et al*. treated drug–disease association prediction as a matrix completion problem and introduced graph regularization to leverage the similarity between drugs and diseases. They employed the parallel proximal algorithm (PPXA) to minimize the objective function and enhance the prediction accuracy of drug–disease interactions [12]. Furthermore, a new weight-regularized matrix factorization method called WRMF was developed by integrating known drug–virus association networks, drug–drug chemical structure similarity networks and virus–viral genome sequence similarity networks. This approach enables the prediction of potential drug–virus associations, thereby improving the accuracy and reliability of inferring potential drugs for new viruses [13]. Despite the promising results achieved by these methods, there are still certain limitations. Firstly, the presence of noise or outliers in the data can significantly impact the performance of these algorithms. Secondly, the selection of appropriate features plays a crucial role in the accuracy of the predictions. Finally, matrix factorization and completion algorithms can be computationally intensive, particularly when dealing with large-scale data, which leads to significant computational complexity.

Deep learning techniques are widely utilized in various areas of bioinformatics [14–17]. Capitalizing on the powerful capabilities of deep learning, researchers have developed several deep learning–based models for drug repositioning. One such model is deepDR, proposed by Zeng *et al*., which integrates diverse information from multiple heterogeneous networks, including drug–drug, drug–disease and drug–target networks. It captures complex topological patterns present in different types of networks [18]. However, deepDR does not differentiate the importance of different nodes, which limits the utilization of information and can impact model performance. To address this limitation, Yu *et al*. introduced the layer-attention graph convolutional network (LAGCN) model [19]. The LAGCN leverages graph convolution operations on embedded heterogeneous networks to learn representations of drugs and diseases. It considers the varying contributions of different embeddings from different layers and employs a layer attention mechanism to combine them into the final representations. However, LAGCN overlooks the varying degrees of correlation between nodes within the same layer, limiting its ability to capture detailed and high-quality node information. In another approach, Sun *et al*. proposed PSGCN, a graph convolutional network–based ‘partner-specific’ method. PSGCN transforms the drug–disease association prediction problem into a graph classification task. It first extracts an h-hop subgraph containing the h-hop neighborhood information of the target drug–disease pair and then performs graph convolution operations on this subgraph. By leveraging rich contextual information, PSGCN introduces finer local structural features to infer potential drug–disease associations [20]. Similarly to LAGCN, PSGCN does not consider the degree of correlation among different nodes. Li *et al*. employed deep convolutional neural networks to learn representations of drugs and diseases using molecular structure and clinical symptom information, respectively, to predict drug–disease associations [21]. However, similar to the previous models, this work overlooks the degree of correlation between node representations. Another model, HINGRL, proposed by Zhao *et al*., integrates different heterogeneous networks with biological knowledge of drugs and diseases to construct heterogeneous information networks. It learns node characteristics from a topological and biological perspective [22]. While this approach enriches the node information and obtains biologically meaningful representations, it ignores potential neighbor information, such as connections between homogeneous nodes. To fully exploit the local topological information of neighborhoods, Meng *et al*. introduced DRWBNCF [23], a computational drug repositioning method based on weighted bilinear neural collaborative filtering. DRWBNCF characterizes the nearest neighbors and their interaction information based on known drug–disease associations, drug–drug similarities and disease–disease similarities. It integrates the known drug–disease associations, drug and disease neighborhoods and neighborhood interaction information into a unified representation. However, DRWBNCF does not consider the weights between different pairs of heterogeneous nodes. The information from different heterogeneous nodes associated with a target node may hold varying importance and should be treated differently.

In this study, we propose a novel drug repositioning method called DRAGNN, based on a graph neural network. Initially, we construct the drug–drug similarity network, disease–disease similarity network and known drug–disease association network to gather heterogeneous information and neighborhood homogeneous information. This enables us to obtain more comprehensive node information. Similar to previous studies, our focus lies in aggregating the information from the top $k$ neighbors to exclude noisy data from the neighborhood. Furthermore, we introduce a graph attention mechanism during the heterogeneous information aggregation process. This mechanism assigns adaptive correlation coefficients to heterogeneous nodes. As a result, the target nodes can aggregate more relevant and valuable information. Differing from previous approaches, DRAGNN does not involve the aggregation of its own node information during the information-gathering process. This prevents excessive information from being embedded within a limited vector space, thereby preserving important heterogeneous and homogeneous information. During the prediction stage, we perform a Hadamard product operation on the drug-embedding vector and the disease-embedding vector to effectively fuse the two sources of information. We then model the complex drug–disease association using a multi-layer perceptron (MLP) to obtain the final association prediction. To evaluate the performance of our model, we compare it with five state-of-the-art methods using three public datasets. Experimental results demonstrate that our model outperforms the others and achieves the highest performance.

## MATERIALS AND METHODS

### Datasets

We evaluated the performance of DRAGNN on three benchmark datasets, which were previously proposed in existing studies. These datasets provide information on the number of drugs, diseases and known drug–disease associations, as shown in Table 1. The first dataset, Fdataset [24, 25], consists of 593 drugs, 313 diseases and 1933 proven drug–disease associations. This dataset corresponds to the work conducted by Gottlieb *et al*. [26]. The drugs were extracted from the comprehensive Drug Bank (DB) database [27], which contains a vast amount of information about drugs and their targets. The diseases were collected from human phenotypes defined in the Online Mendelian Inheritance of Man (OMIM) database [28], which is a publicly available resource providing information on human genes and diseases. The second dataset, Cdataset [29], includes 2352 known associations between 663 drugs from the DrugBank database and 409 diseases from the OMIM database. The third dataset, LRSSL, comprises 763 drugs, 681 diseases and 3051 drug–disease associations [30]. These benchmark datasets serve as valuable resources for evaluating the performance of DRAGNN.

**Table 1:** Basic information of the three public datasets used in this study

Datasets	No. of drugs	No. of diseases	The known associations
Fdataset	593	313	1933
Cdataset	663	409	2532
LRSSL	763	681	3051

### The construction of three networks

We begin by constructing three networks: a drug–drug similarity network, a disease–disease similarity network and a known drug–disease association network. To represent the known drug–disease association network $G$, we utilize a binary matrix $A\in{\mathbb{R}}^{n\ast m}$; $n$ and $m$ in the matrix represent the number of drugs and diseases, respectively. Each element ${A}_{ij}\in \left\{0,1\right\}$ in the matrix corresponds to a specific drug ${r}_i$ and disease ${d}_j$. If the drug ${r}_i$ and disease ${d}_j$ are confirmed to be related, ${A}_{ij}=1$; otherwise, ${A}_{ij}=0$.

The drug–drug similarity network, denoted as ${G}^r$, can be represented by a matrix ${A}^r\in{\mathbb{R}}^{n\ast n}$, where each entry in ${A}^r$ is constructed based on the similarity information between drug pairs. Specifically, the drug similarity can be represented by an $n\times n$ square matrix ${\mathrm{S}}^r$, where ${\mathrm{S}}^r\left(i,j\right)$ denotes the similarity between drug ${r}_i$ and drug ${r}_j$. To mitigate the influence of noisy information on node representation learning, we concentrate on aggregating the information from the top $k$ neighbors. When two nodes belong to the top $k$ neighbors, their corresponding value is set to 1 instead of their similarity score. This is done to avoid insufficient utilization of neighbor information due to low similarity and achieve the effect of enhancing neighbor information. Consequently, the entry ${A}_{ij}^r$ in ${A}^r$ can be defined as follows:


(1) \begin{equation*} {A}_{ij}^r=\left\{\begin{array}{l} 1\kern2em \mathrm{if}\ {r}_j\in{N}_k\left({r}_i\right)\\{}0\kern2em \mathrm{otherwise}\end{array}\right. \end{equation*}

${N}_k\left({r}_i\right)$ represents the first $k$ neighbors corresponding to the drug ${r}_i$, which are obtained from ${\mathrm{S}}^r$. These neighbors are the $k$ drugs with the highest similarity to drug ${r}_i$, excluding ${r}_i$ itself. Similarly, the disease–disease similarity network ${G}^d$ can be represented by ${A}^d\in{\mathbb{R}}^{m\ast m}$, where each entry in ${A}^d$ is constructed based on the similarity information of each pair of diseases. Disease similarity can be expressed as a square matrix ${\mathrm{S}}^d$ of size $m\times m$, where ${\mathrm{S}}^d\left(i,j\right)$ represents the similarity between disease ${d}_i$ and disease ${d}_j$. Similarly, each entry ${A}_{ij}^d$ in ${A}^d$ can be defined as follows:


(2) \begin{equation*} {A}_{ij}^d=\left\{\begin{array}{l} 1\kern2em \mathrm{if}\ {d}_j\in{N}_k\left({d}_i\right)\\ {}0\kern2em \mathrm{otherwise}\end{array}\right. \end{equation*}

where ${N}_k\left({d}_i\right)$ represents the first $k$ neighbors corresponding to disease ${d}_i$, which are obtained based on ${\mathrm{S}}^d$. Similarly, ${N}_k\left({d}_i\right)$ does not include ${d}_i$.

### Model architecture

**Figure 1:**
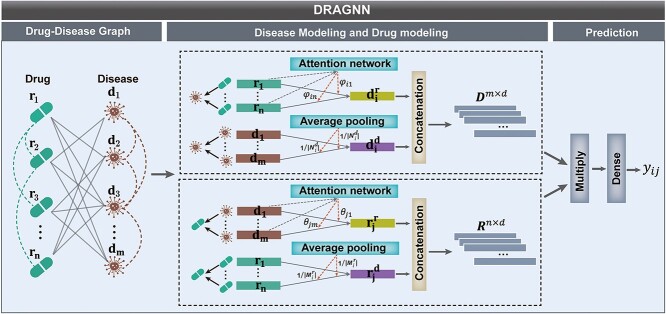
The overall architecture of DRAGNN. The constructed drug–disease heterogeneous network serves as the input to the model. The disease modeling involves two steps: heterogeneous information aggregation and neighbor information aggregation. In the process of heterogeneous information aggregation, a graph attention mechanism is introduced to adaptively assign different weights to different heterogeneous nodes, allowing the target node to aggregate more effective information. As for neighbor information aggregation, average pooling is used to ensure a concise aggregation process while preserving the effect of enhancing neighbor information. By concatenating the aggregated heterogeneous information and neighbor information, the final representation of the disease is obtained. The process of drug modeling is similar to the disease modeling, leading to the final representation of the drug. During the prediction phase, the Hadamard product is first used to fully integrate the drug and disease representations. Subsequently, an MLP is applied to model the complex drug–disease associations, resulting in the final prediction.

The overall architecture of DRAGNN is shown in Figure 1. In the following section, we will elaborate on the primary modules of the model. For ease of reference, lowercase bold letters will represent vectors, while uppercase bold letters will denote matrices.

#### Disease modeling

For each disease ${d}_i$, disease modeling learns the corresponding latent vector representation ${\boldsymbol{d}}_i$ by aggregating two types of information: drug–disease interaction information (denoted as ${\boldsymbol{d}}_i^r$) and disease–disease interaction information (denoted as ${\boldsymbol{d}}_i^d$).


(3) \begin{equation*} {\boldsymbol{d}}_i={\boldsymbol{d}}_i^r+{\boldsymbol{d}}_i^d \end{equation*}

Specifically, the heterogeneous information is aggregated by means of the drug nodes associated with the disease node.


(4) \begin{equation*} {\boldsymbol{d}}_i^r=\sigma \left(\boldsymbol{W}\left(\sum_{j\in{N}_i^r}{\varphi}_{ij}{\boldsymbol{r}}_j\right)+\boldsymbol{b}\right) \end{equation*}

where ${\boldsymbol{r}}_j$ represents the embedding vector of drug ${r}_j$, ${\varphi}_{ij}$ is the interaction coefficient between ${d}_i$ and ${r}_j$ and ${N}_i^r$ represents the set of drug nodes directly related to ${d}_i$. $\sigma$ denotes an activation function, while $\boldsymbol{W}$ and $\boldsymbol{b}$ represent a weight matrix and a bias vector, respectively.

Homogeneous information is aggregated based on the top $k$ neighboring nodes (disease nodes) of the disease node.


(5) \begin{equation*} {\boldsymbol{d}}_i^d=\sigma \left(\boldsymbol{W}\left(\frac{\sum_{j\in{N}_i^d}{\boldsymbol{d}}_j}{\left|{N}_i^d\right|}\right)+\boldsymbol{b}\right) \end{equation*}

where ${N}_i^d$ represents the first $k$ neighboring disease nodes of ${d}_i$.

There are two approaches to calculate ${\varphi}_{ij}$. The first approach assumes equal contributions from all nodes to the target node, i.e. ${\varphi}_{ij}=\frac{1}{\left|{N}_i^r\right|}$. However, this method may not be optimal, as nodes with similar characteristics are better suited for modeling to extract more effective information. Therefore, the second approach assigns different contributions to different nodes. In this study, we employ the graph attention mechanism [31] to enable disease/drug nodes to pay more attention to interactions with nodes that exhibit a high degree of correlation.


(6) \begin{equation*} {\varphi}_{ij}={\boldsymbol{w}}_2^T\sigma \left({\boldsymbol{W}}_1\left[{\boldsymbol{r}}_j\oplus{\boldsymbol{d}}_i\right]+{\boldsymbol{b}}_1\right)+{b}_2 \end{equation*}

Here, $\oplus$ refers to the concatenation operation, and we utilize the softmax function to normalize the coefficients, which can be interpreted as determining the importance of ${r}_j$ to the latent vector representation of ${d}_i$.

To aggregate neighbor information for the disease node effectively while maintaining a simple approach, a straightforward average pooling method is employed, ensuring the retention of the enhancement effect from the neighbor nodes’ information, as shown in Equation (5).

#### Drug modeling

Similarly, for each drug ${r}_i$, drug modeling learns the corresponding latent vector representation by aggregating disease–drug interactions (denoted as ${\boldsymbol{r}}_i^d$) and drug–drug interactions (denoted as ${\boldsymbol{r}}_i^r$).


(7) \begin{equation*} {\boldsymbol{r}}_i={\boldsymbol{r}}_i^d+{\boldsymbol{r}}_i^r \end{equation*}

Here, the heterogeneous information is aggregated using the disease nodes associated with the drug node.


(8) \begin{equation*} {\boldsymbol{r}}_i^d=\sigma \left(\boldsymbol{W}\left(\sum_{j\in{M}_i^d}{\theta}_{ij}{\boldsymbol{d}}_j\right)+\boldsymbol{b}\right) \end{equation*}

where ${\boldsymbol{d}}_j$ represents the embedding vector of drug ${d}_j$, ${\theta}_{ij}$ is the interaction coefficient between ${r}_i$ and ${d}_j$ and ${M}_i^d$ represents the set of disease nodes directly related to ${r}_i$. $\sigma$ is the activation function, while $\boldsymbol{W}$ and $\boldsymbol{b}$ represent the weight matrix and bias vector, respectively.

Homogeneous information is aggregated based on the top $k$ neighboring nodes (drug nodes) of the drug node, average pooling is also employed here.


(9) \begin{equation*} {\boldsymbol{r}}_i^r=\sigma \left(\boldsymbol{W}\left(\frac{\sum_{j\in{M}_i^r}{r}_j}{\left|{M}_i^r\right|}\right)+\boldsymbol{b}\right) \end{equation*}

where ${M}_i^r$ represents the top $k$ neighboring drug nodes of ${r}_i$.

Similarly, we utilize the graph attention mechanism to define ${\theta}_{ij}$.


(10) \begin{equation*} {\theta}_{ij}={\boldsymbol{w}}_2^T\sigma \left({\boldsymbol{W}}_1\left[{\boldsymbol{d}}_j\oplus{\boldsymbol{r}}_i\right]+{\boldsymbol{b}}_1\right)+{b}_2 \end{equation*}

Here, softmax is used to normalize the interaction coefficients in this case as well.

#### MLP-based prediction

For drug ${r}_i$ and disease ${d}_j$, we first multiply them to obtain ${\boldsymbol{g}}_0=\left[{\boldsymbol{r}}_i\cdot{\boldsymbol{d}}_j\right]$, then pass it through an MLP and finally apply the sigmoid function to obtain the association probability score ${y}_{ij}$ for ${r}_i$ and ${d}_j$.

${\boldsymbol{g}}_1=\sigma \left({\boldsymbol{W}}_0{\boldsymbol{g}}_0+{\boldsymbol{b}}_0\right)$ ，.

${\boldsymbol{g}}_2=\sigma \left({\boldsymbol{W}}_1{\boldsymbol{g}}_1+{\boldsymbol{b}}_1\right)$ ，


$$ \cdots \cdots $$

${\boldsymbol{g}}_l=\sigma \left({\boldsymbol{W}}_{l-1}{\boldsymbol{g}}_{l-1}+{\boldsymbol{b}}_{l-1}\right)$ ，


(11) \begin{equation*} {y}_{ij}=\mathrm{sigmoid}\left({W}_l^T{\boldsymbol{g}}_l+{\boldsymbol{b}}_l\right) \end{equation*}

where $l$ is the number of hidden layers.

#### Model training

We define the loss function as follows:


(12) \begin{equation*} \mathrm{loss}=-\sum_{i=1}^n\sum_{j=1}^m\left[{y}_{ij}^{\ast}\log \left({y}_{ij}\right)+\left(1-{y}_{ij}^{\ast}\right)\log \left(1-{y}_{ij}\right)\right] \end{equation*}

where ${y}_{ij}^{\ast }$ is the true label between drug ${r}_i$ and disease ${d}_j$ and $n$ and $m$ represent the number of drugs and diseases, respectively. To optimize this loss function, we utilize the Adam optimizer [32] and cyclic learning rate [33].

## EXPERIMENT

### Evaluation metrics

To evaluate the performance of DRAGNN, we utilized a 10-fold cross-validation approach. This involved dividing the positive and negative samples equally into 10 folds, where in each fold, one set of positive and negative samples was used as the test set, while the remaining data were used for training our model. The area under the ROC curve (AUROC) and the area under the precision–recall curve (AUPR) are widely used metrics in bioinformatics research [2, 23] and are employed to assess the overall performance of DRAGNN.

### Baseline methods

To showcase the superiority of the DRAGNN model, we conducted a comparison with five advanced models on three datasets: Fdataset, Cdataset and LRSSL. The models included in the comparison were DRWBNCF [23], LBMFF [34], SCMFDD [35], SCPMF [8] and HNRD [36].

DRWBNCF, by employing a novel weighted bilinear graph convolution operation, the local information of different networks is integrated into a unified representation. Finally, an MLP optimized with $\mathrm{\alpha}$ balanced focal loss function and graph regularization is utilized to model complex drug–disease associations.

LBMFF is a drug–disease association prediction method known as Literature-Based Multi-Feature Fusion. Firstly, it effectively integrates known associations of drugs, diseases, side effects and targets from public databases, along with semantic features from the literature. Secondly, it incorporates a pre-trained and fine-tuned BERT model to extract semantic information from the literature for similarity assessment. Finally, drug and disease embeddings are uncovered from the constructed fusion similarity matrix using a graph convolutional network with an attention mechanism.

SCMFDD is a similarity-constrained matrix factorization method used for drug–disease association prediction. It projects the association relationships between drugs and diseases into two low-dimensional spaces, revealing the latent features of drugs and diseases. It then introduces similarity based on drug features and disease semantic similarity as constraints in the low-dimensional space for drugs and diseases.

SCPMF is applied to the adjacency matrix of a heterogeneous drug–virus network, which incorporates known drug–virus interactions, drug chemical structures and viral genome sequences. SCPMF projects the drug–virus interaction matrix onto two latent feature matrices for drugs and viruses and reconstructs the drug–virus interaction matrix through matrix multiplication. Moreover, it introduces a weighted similarity interaction matrix as a constraint for drugs and viruses.

HNRD is a model for predicting the associations between drugs and diseases. It relies on neighborhood information aggregation in neural networks and combines the similarity of diseases and drugs with their associations.

### Parameters setting

For our model, DRAGNN, in all experiments, we set the initial learning rate to 0.01, the maximum training epoch to 15 and the node dimension to 125. Regarding the number of neighbors, we select 7 from the range of [1, 2, 3, … , 20], and the number of MLP layers is set to 1. The hyperparameters of the comparison models are chosen based on the optimal values suggested by the original authors.

## RESULTS AND DISCUSSIONS

### Performance of DRAGNN in 10-fold cross-validation

To evaluate the performance of DRAGNN, we compared it with baseline models on three datasets. Table 2 presents the results of our model using 10-times 10-fold cross-validation, alongside other models. DRAGNN outperforms all comparison models across the three datasets, achieving higher overall performance in two evaluation metrics. The average AUROC and AUPR are 0.947 and 0.571, respectively, which are 1.4% and 3.8% higher than the second-best performance. Supplementary Figure S1 available online at http://bib.oxfordjournals.org/ illustrates the corresponding ROC curve and PR curve. Notably, the AUPR value significantly surpasses the other models, indicating that our utilization of the graph attention mechanism enables us to aggregate more effective heterogeneous information during node representation learning. Additionally, to prioritize the significance of neighborhood information and heterogeneous information, we opted to exclude the aggregation of self-node information. This approach allowed the node to obtain high-quality node representations, leading to improved prediction performance.

**Table 2:** The AUROCs and AUPRs of DRAGNN and the other five comparative models under 10-times 10-fold cross-validation

Datasets	LBMFF	SCMFDD	SCPMF	HNRD	DRWBNCF	DRAGNN
AUROCs						
Fdataset	0.81642 ± 0.035	0.77615 ± 0.001	0.89380 ± 0.001	0.88070 ± 0.004	0.92469 ± 0.001	**0.94415 ± 0.002**
Cdataset	0.90798 ± 0.001	0.79317 ± 0.001	0.91316 ± 0.002	0.90383 ± 0.003	0.94064 ± 0.001	**0.94786 ± 0.007**
LRSSL	0.91053 ± 0.002	0.76840 ± 0.001	0.89565 ± 0.001	0.84933 ± 0.003	0.93596 ± 0.001	**0.95039 ± 0.005**
Avg.	0.87831	0.76840	0.90087	0.87795	0.93376	**0.94746**
AUPRs						
Fdataset	0.12052 ± 0.012	0.00557 ± 0.000	0.34972 ± 0.006	0.53932 ± 0.007	0.49132 ± 0.006	**0.59892 ± 0.025**
Cdataset	0.20823 ± 0.006	0.00558 ± 0.000	0.42327 ± 0.004	**0.63121 ± 0.005**	0.56828 ± 0.004	0.61437 ± 0.025
LRSSL	0.16960 ± 0.005	0.00379 ± 0.000	0.27112 ± 0.002	0.42845 ± 0.004	0.35215 ± 0.005	**0.49971 ± 0.021**
Avg.	0.16614	0.00498	0.34803	0.53299	0.47058	**0.57100**

*Note.* The best reported result is bolded, and the second-best result is underlined.

**Figure 2:**
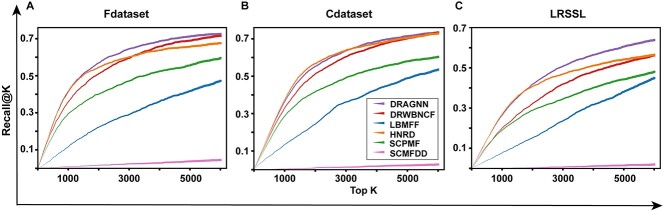
The Recall@k values against the top k predicted list of DRAGNN and other compared methods during 10-fold cross-validation on (**A**) Fdataset, (**B**) Cdataset and (**C**) LRSSL, respectively.

Taking into account the severe imbalance between the number of positive and negative associations, similar to Zeng *et al*., we utilize the recall@k metric [18] to further assess our model. The recall@k metric measures the proportion of correctly identified positive associations among all positive associations in the dataset for the first $k$ predictions. It represents the ratio of the number of positive associations correctly identified in the top $k$ predictions to the total number of positive associations in the dataset. As shown in Figure 2, our model outperforms all other models in terms of recall@k for the first 6000 predictions on the three datasets.

### Discovering candidates for new diseases

In order to assess DRAGNN’s predictive capabilities for new diseases, we conducted a *de novo* test on the Cdataset, following a methodology similar to previous studies [2]. For this test, we excluded all known drug associations, using them as the test set, and utilized the remaining associations as training samples. As illustrated in Supplementary Table S1 available online at http://bib.oxfordjournals.org/, DRAGNN yielded the most favorable results, with an AUROC value of 0.77748 and an AUPR value of 0.09977, thereby showcasing DRAGNN’s potential in the prediction of drug candidates for new diseases.

### Ablation analysis

The advantage of our model lies in the incorporation of local neighborhood information during the node-embedding learning process. Additionally, to ensure the quality of information and simplicity of the aggregation process, we exclude the consideration of a node’s own information during the node update process. Furthermore, the graph attention mechanism is used to assign different weights to various heterogeneous nodes, enabling more effective information aggregation by the nodes. Finally, for a specific drug–disease pair, we perform element-wise multiplication on their respective representation vectors, followed by modeling the complex drug–disease association using an MLP to obtain the final prediction result. Therefore, six variants of DRAGNN are proposed and summarized as follows:

DRAGNN-noNei: During the information aggregation process, neither the local neighborhood information of drugs nor the local neighborhood information of diseases is taken into consideration.

DRAGNN-disNei: In the process of information aggregation, only the local neighborhood information of the disease is considered, while the local neighborhood information of the drug is not taken into account.

DRAGNN-drNei: In the process of information aggregation, only the local neighborhood information of the drug is considered, while the local neighborhood information of the disease is not taken into account.

DRAGNN-noMLP: After performing the element-wise multiplication of the representation vectors corresponding to the two nodes, the final prediction is obtained through a simple fully connected layer, instead of using an MLP.

DRAGNN-selfAg: In the node information update process, we consider not only the heterogeneous information and neighborhood information but also the original information of the node itself.

DRAGNN-noAtt: In the process of aggregating heterogeneous information for the target node, equal importance is assigned to different heterogeneous node information.

The performance of DRAGNN and its six variants on three datasets is shown in Table 3. The performance of DRAGNN-noNei is notably reduced compared to DRAGNN, highlighting the importance of incorporating neighborhood information in node representation learning. Moreover, both DRAGNN-disNei and DRAGNN-drNei exhibit decreased performance compared to DRAGNN, indicating the indispensability of both drug neighbor information and disease neighbor information for enhancing the performance of DRAGNN. It is noteworthy that DRAGNN-disNei outperforms DRAGNN-drNei significantly on the three datasets. One possible explanation is the presence of more ‘popular entities’ [37] among the disease neighbor nodes, referring to disease nodes with a greater number of known associations. These disease nodes contain abundant information that can contribute effectively when they are involved in the modeling process as neighbors. Consequently, this leads to the generation of high-quality node representations and ultimately improves the prediction performance. The two metrics of DRAGNN-noMLP on the three datasets are inferior to those of DRAGNN. This disparity can be attributed to the removal of the MLP component, which compromises the model’s ability to capture the complex relationship between diseases and drugs during the prediction stage, resulting in suboptimal performance. The performance of DRAGNN-selfAg is also significantly lower than that of DRAGNN due to the introduction of more noisy information through self-aggregation. This dilutes the useful neighborhood information and heterogeneous information, ultimately reducing the quality of the final representations and impacting overall model performance. Compared to DRAGNN, DRAGNN-noAtt exhibits a notable drop in performance because it considers the correlation coefficients corresponding to different node information as equal, thereby introducing more invalid information that impairs the final performance.

**Table 3:** The AUROCs and AUPRs of DRAGNN and its six variants under 10-fold cross-validation

Variants	Fdataset		Cdataset		LRSSL	
	AUROC	AUPR	AUROC	AUPR	AUROC	AUPR
DRAGNN-noNei	0.615	0.129	0.610	0.126	0.622	0.042
DRAGNN-disNei	0.873	0.491	0.864	0.469	0.895	0.406
DRAGNN-drNei	0.713	0.211	0.794	0.289	0.808	0.251
DRAGNN-selfAg	0.853	0.251	0.886	0.378	0.848	0.268
DRAGNN-noAtt	0.736	0.122	0.761	0.149	0.777	0.139
DRAGNN-noMLP	0.906	0.522	0.930	0.541	0.944	0.455
DRAGNN	0.951	0.629	0.955	0.630	0.950	0.508

### The effect of different hyperparameters

In this section, we will examine the influence of various hyperparameters on the performance of the model, with the aim of uncovering the underlying factors that affect its performance.

**Figure 3:**
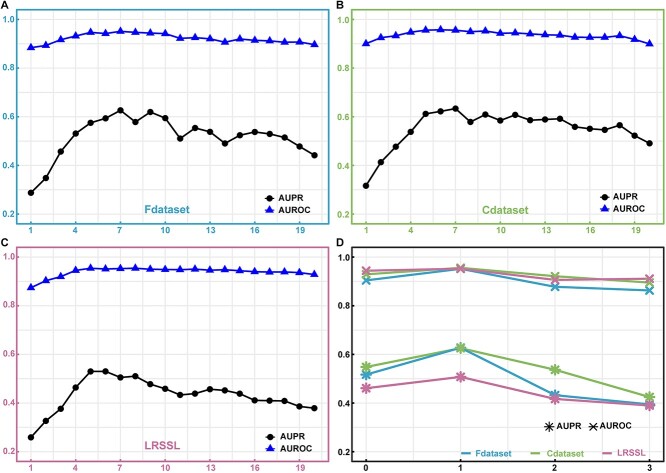
The performance of DRAGNN using 10-fold cross-validation with different hyperparameters. (**A**), (**B**) and (**C**) represent the model’s performance corresponding to different numbers of neighbors on the Fdataset, Cdataset and LRSSL, respectively. (**D**) shows the performance of the model at different numbers of MLP layers.

#### The number of nearest neighbors

One of the key factors contributing to the performance improvement of our model is the integration of high-quality neighborhood information through top-*k* neighbors. Therefore, selecting an appropriate number of neighbors is crucial. Figure 3A–C illustrates the AUROC and AUPR values corresponding to the number of neighbors in the range of [1, 20], on Fdataset, Cdataset and LRSSL, respectively. We observe that for all three datasets, the overall trend of AUROC and AUPR is an initial increase followed by a decrease as the number of neighbors increases. When the number of neighbors is small, the acquired neighborhood information during the representation learning process is limited, thereby constraining the model’s performance. As the number of neighbors increases, the abundance of neighborhood information improves, leading to enhanced representation quality of the nodes and consequently improving the model’s performance up to an optimal value. However, as the number of neighbors continues to increase, the inclusion of neighbor node information with low similarity to the target node introduces some irrelevant information, thereby diminishing the representation quality of the target node. Consequently, the model’s performance gradually deteriorates. Notably, the model achieves the best overall performance on the three datasets when the number of neighbors is set to 7.

**Table 4:** The top 10 DRAGNN-predicted candidate drugs for PD

Rank	Candidate drugs (DrugBank IDs)	Evidence
1	Clonazepam (DB01068)	ClinicalTrials.gov
2	Haloperidol (DB00502)	DrugCentral
3	Galantamine (DB00674)	ClinicalTrials.gov
4	Vitamin E (DB00163)	CTD, PubChem, ClinicalTrials.gov
5	Dantrolene (DB01219)	[39]
6	Rivastigmine (DB00989)	DB, CTD, PubChem, DrugCentral, ClinicalTrials.gov
7	Dopamine (DB00988)	DB, ClinicalTrials.gov
8	Ziprasidone (DB00246)	[40]
9	Phenylpropanolamine (DB00397)	NA
10	Memantine (DB01043)	DB, PubChem, ClinicalTrials.gov

**Table 5:** The top 10 DRAGNN-predicted candidate drugs for BC

Rank	Candidate drugs (DrugBank IDs)	Evidence
1	Doxorubicin (DB00997)	DB, ClinicalTrials.gov
2	Cisplatin (DB00515)	DB, ClinicalTrials.gov
3	Docetaxel (DB01248)	DB, DrugCentral, ClinicalTrials.gov
4	Methotrexate (DB00563)	DB, ClinicalTrials.gov
5	Vincristine (DB00541)	DB
6	Bleomycin (DB00290)	NA
7	Dinoprostone (DB00917)	NA
8	Tretinoin (DB00755)	[43]
9	Teniposide (DB00444)	[42]
10	Paclitaxel (DB01229)	DB, ClinicalTrials.gov

**Table 6:** The molecular binding energies between the top 10 DRAGNN-predicted candidate drugs for PD and five target proteins (kcal/mol)

Drugs	The molecular binding energies between the drugs and five target proteins (kcal/mol)				
	1j42	1lcy	2pzd	4zgg	6xaf
Clonazepam	−9.3	−10.2	−8.6	−8.5	−8.5
Haloperidol	−8.3	−10.5	−8.4	−7.9	−8.7
Galantamine	−8.5	−9.8	−8.5	−8.6	−8.8
Vitamin E	−7.5	−10.5	−7.2	−6.6	−8.1
Dantrolene	−7.3	−10.1	−7.6	−8.1	−8.0
Rivastigmine	−6.1	−7.2	−6.1	−6.0	−5.9
Dopamine	−5.7	−6.8	−5.2	−5.4	−6.0
Ziprasidone	−8.4	−11.9	−9.1	−8.8	−8.9
Phenylpropanolamine	−6.1	−6.0	−5.3	−5.6	−6.0
Memantine	−8.1	−8.9	−7.6	−8.0	−8.3

#### The layers of MLP

In the prediction stage, an MLP is used to model the complex relationship between drugs and diseases. To evaluate the impact of MLP layers on the model’s performance, we conducted a study with different numbers of MLP layers: 0, 1, 2 and 3. When the MLP has 0 layers, only a simple fully connected layer is used to obtain the prediction results. As shown in Figure 3D, the results clearly demonstrate that the model’s performance is suboptimal when relying solely on a simple fully connected layer without an MLP. This is because a simple fully connected layer lacks the capacity to capture complex associations, thus limiting the model’s expressive power. On the other hand, when the number of MLP layers is set to 1, the performance reaches its optimum. The addition of an MLP introduces non-linear transformations that enable the model to learn more intricate feature representations. However, as the number of MLP layers increases beyond 1, both AUROC and AUPR experience a significant decline. This can be attributed to the heightened complexity and computational burden of the model, as well as the increased risk of overfitting. Consequently, the model’s generalization ability is compromised, leading to a decrease in performance.

**Figure 4:**
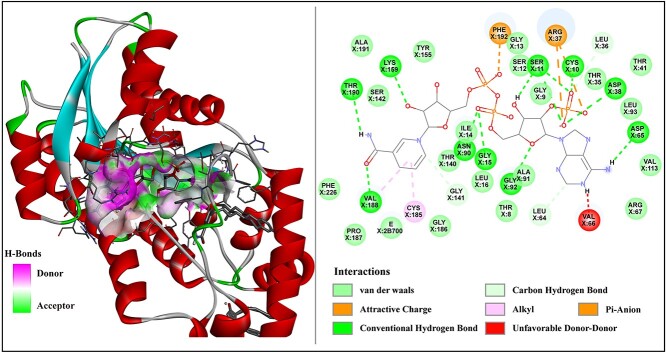
3D and 2D diagrams of molecular docking results. The molecular docking results clearly show that there are van der Waals interactions between the small molecule and 20 amino acid residues, including Ala191, Tyr155 and Ser142. Additionally, conventional hydrogen bond interactions are observed between the small molecule and 10 amino acid residues, such as Lys159, Thr190 and Val188. Furthermore, the small molecule exhibits carbon–hydrogen bond interactions with Gly141, Leu64 and Leu36 and pi–anion interactions with Phe192 and Arg37. There is also an alkyl interaction between the compound and the residue Cys185.

### Case studies

To validate the applicability of DRAGNN in practical scenarios, we performed case studies to predict drug candidates for two diseases: Parkinson’s disease (PD) and breast cancer (BC). For these predictions, we trained our model using all known drug–disease associations in the Fdataset, while the unknown drug–disease associations were considered as candidate sets. After obtaining the probabilities for all drug–disease associations, the drugs were sorted in descending order based on their probabilities. Subsequently, we selected the top 10 candidate drugs for each disease for further investigation. We utilized authoritative data sources, namely, DB, CTD, PubChem, DrugCentral and ClinicalTrials, to verify the accuracy of DRAGNN’s prediction results.

PD is a progressive disease that affects the nervous system and various parts of the body controlled by nerves. While there is no cure for PD, medications can significantly alleviate symptoms. Galantamine, commonly used to treat cognitive decline in mild to moderate Alzheimer’s disease and other memory impairments, has been predicted as a potential drug for PD. Previous studies have demonstrated the usefulness of cholinesterase inhibitors with additional nicotinic activity, such as galantamine, in Parkinsonian patients with dementia [38]. This association is further supported by ClinicalTrials. Vitamin E, an essential nutrient for vision; reproduction; and overall brain, blood and skin health, is predicted by DRAGNN to have an association with PD. This prediction aligns with evidence from CTD, PubChem and ClinicalTrials, as a deficiency in vitamin E can lead to neuropathy. Additionally, rivastigmine and memantine, both predicted by DRAGNN, have been confirmed by DB, CTD, PubChem, DrugCentral and ClinicalTrials as treatments for PD. In summary, 9 out of the top 10 predicted drugs with probability scores (a success rate of 90%) as shown in Table 4 have been validated by reliable sources, clinical trials and published studies.

BC is characterized by uncontrolled proliferation of breast epithelial cells influenced by various carcinogenic factors, making it the most prevalent malignancy among women. DRAGNN predicts doxorubicin, a DNA-targeting drug widely used in chemotherapy, as the top potential treatment for BC. This prediction is supported by evidence from DB and ClinicalTrials. Previous studies have emphasized the efficacy of liposomal doxorubicin in treating metastatic and early-stage breast cancer, solidifying its role as a cornerstone of breast cancer therapy [41]. Methotrexate, a chemotherapy and immunosuppressant drug employed in the management of cancer, autoimmune diseases, ectopic pregnancy and medical abortion, is also predicted as a potential treatment for BC, with validation from DB and ClinicalTrials. Teniposide, widely used in the treatment of small cell lung cancer, malignant lymphoma and breast cancer [42], is identified as a candidate drug for BC by DRAGNN. Additionally, cisplatin and docetaxel, predicted by DRAGNN, are confirmed by DB, DrugCentral and ClinicalTrials for their effectiveness in BC treatment. As Table 5 demonstrates, 8 out of the top 10 predicted drugs with probability scores (a success rate of 80%) have been substantiated by reliable sources, clinical trials and published studies.

In addition, we selected five target proteins corresponding to PD and used AutoDock Vina [44] to perform molecular docking experiments with 10 candidate drugs specific to PD. The corresponding molecular binding energies were obtained for each target protein and its respective candidate drug. In molecular docking experiments, the molecular binding energy serves as an indicator of the strength of binding between the ligand (drug molecule) and the receptor (target protein). A higher absolute value of the molecular binding energy indicates a stronger affinity of the ligand for the target receptor. As shown in Table 6, for the unvalidated predicted candidate drug phenylpropanolamine, the molecular binding energies with the target proteins having PDB codes 1j42, 1lcy, 2pzd, 4zgg and 6xaf are −6.1, −6.0, −5.3, −5.6 and −5.0 kcal/mol, respectively. The molecular binding energies between the other nine candidate drugs and different target proteins are also presented in Table 6. The molecular binding energies of the five target proteins corresponding to BC and the 10 candidate drugs are shown in Supplementary Table S2 available online at http://bib.oxfordjournals.org/.

We used DS software to visualize the docking results of dinoprostone and estradiol (PDB code: 3HB5). The non-covalent and hydrophobic interactions between drugs and proteins facilitate the binding of drugs to specific sites on proteins, thereby exerting their medicinal effects. As shown in Figure 4, there are van der Waals interactions and conventional hydrogen bond interactions between small molecules and amino acid residues, which are common non-covalent interactions. Additionally, there are some hydrophobic interactions, such as carbon–hydrogen bond interactions, pi–anion interactions and alkyl interactions. The visualization results indicate that the drug candidates corresponding to BC, as predicted by our model, may provide valuable references for clinicians.

**Figure 5:**
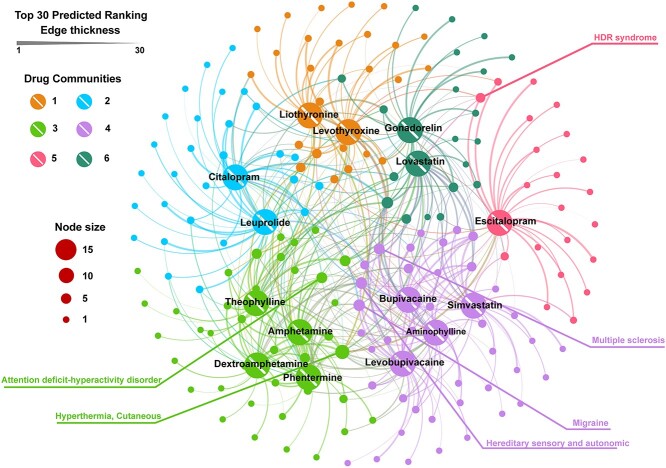
Network diagram of 15 drugs and 174 diseases. Community 1 contains liothyronine and levothyroxine, both prescribed to treat hypothyroidism. Community 2 is composed of citalopram and leuprolide, where citalopram improves the state of mind by increasing serotonin concentration in the brain and leuprolide belongs to the gonadotropin-releasing hormone class. Community 3 includes theophylline, amphetamine, dextroamphetamine and phentermine. Both amphetamine and dextroamphetamine can be used to treat ADHD. Community 4 comprises bupivacaine, simvastatin, aminophylline and levobupivacaine. Bupivacaine and levobupivacaine are anesthetic drugs, simvastatin reduces cardiovascular risk and aminophylline treats bronchospasm. Escitalopram represents community 5, and gonadorelin and lovastatin represent community 6. In the network representation, each disease or drug is depicted as a node, with its size determined by the node’s degree. The thicker the line in the figure, the higher the predicted ranking between the two nodes.

Based on the drug similarity matrix in the Fdataset, we selected 15 drugs corresponding to the top 10 similar drug–drug pairs. Utilizing DRAGNN, we predicted 30 candidate diseases for each drug, resulting in a total of 174 diseases. The association network between these 15 drugs and 174 diseases is displayed in Figure 5. Employing the modular function in Gephi software, we categorized the 15 drugs into six distinct communities.

Hyperthermia, the drug node with the highest degree, was predicted to be associated with 13 out of the 15 drugs, excluding gonadorelin and simvastatin. Among the 15 drugs, 8 were predicted to have a connection with attention-deficit hyperactivity disorder (ADHD), which is the most common childhood-onset behavioral disorder. This set includes amphetamine and dextroamphetamine, both of which belong to community 3 and are used to treat ADHD. Regarding migraine, 9 out of the 15 drugs were predicted to be associated with it, including bupivacaine and levobupivacaine, both anesthetic drugs from community 4. A previous study also discussed the use of narcotic analgesics in the emergency treatment of migraine [45]. Additionally, the diseases hereditary sensory and autonomic, multiple sclerosis and HDR syndrome were each predicted to be associated with nine, eight and six drugs, respectively.

To sum up, the analysis results demonstrate that our model can accurately predict some of the disease–drug community associations. This indicates that even with a large amount of data, DRAGNN can effectively identify certain communities for different drugs and potentially offer new insights for the exploration of drug combinations in the future.

## CONCLUSION

In this study, we present DRAGNN, a deep learning–based computational method for drug repositioning. DRAGNN incorporates neighborhood information during the node representation learning process, thereby preserving local topological context information. When constructing the drug–drug similarity matrix and the disease–disease similarity matrix, the values between the top $k$ neighbors are set to 1 instead of their similarity values. This avoids the similarity being too small to fully utilize the neighbor information, thus achieving the effect of neighbor information enhancement. At the same time, in the process of node representation learning, the aggregation of self-node information is discarded, which emphasizes the role of heterogeneous information and neighbor information more, ultimately enhancing local information. We carefully control the number of neighbors to ensure the utilization of effective neighborhood information while mitigating the impact of invalid or noisy neighborhood information, leading to improved prediction performance. This approach serves as a valuable complement to the neighborhood information fusion process. Furthermore, we introduce a graph attention mechanism that plays a role in modeling heterogeneous information of drugs and diseases. This mechanism enables us to differentiate the contributions of different heterogeneous nodes to the target node during the modeling process, facilitating the learning of higher-quality characterizations. By integrating heterogeneous information and neighborhood information into a unified representation, we feed it into an MLP to model complex associations and capture different types of information, ultimately obtaining prediction results. To assess the performance of DRAGNN, we conducted extensive experiments on three benchmark datasets and compared its results with five state-of-the-art association prediction models. The experimental results demonstrated the effectiveness of DRAGNN in achieving superior performance. Additionally, we explored six variants of DRAGNN, examining the impact of neighborhood information, self-aggregation, MLP and attention mechanism on model performance. Furthermore, we investigated two crucial hyperparameters, namely, the number of neighbors and the layer of MLP, to unveil their influence on model performance. These analyses shed light on the underlying factors influencing the model’s performance. The final case study and network analysis experiments demonstrate the robust practical predictive capability of DRAGNN.

While DRAGNN demonstrates strong performance, there are certain limitations to be acknowledged. One limitation is the lack of consideration for multifaceted similarities between drugs or diseases, which could result in underutilization of similarity information. Additionally, exploring the fusion of multiple datasets for training and prediction is an area that warrants further investigation.

**Key Points**

- We propose a deep learning method named DRAGNN for drug repositioning. This method is based on the graph neural network and incorporates weighted local information augmentation.
- We introduce the graph attention mechanism to discern the relevance between distinct heterogeneous node information of drugs and diseases, aiming to learn high-quality embeddings for both drugs and diseases.
- During the information aggregation process, we have omitted the self-node information aggregation step and utilized a fixed value instead of similarity scores to define the aggregation coefficient for neighboring node information. This modification ensures the full utilization of neighbor information and achieves the desired local information augmentation.
- The molecular docking experiment and the network analysis experiment both showcase the potential application of our model in predicting candidate drugs and drug combinations.

## Supplementary Material

supplementary_bbad431

## Data Availability

The implementation of DRAGNN and the preprocessed data is available at https://github.com/1yiw/DRAGNN.

## Author Biographies

**Yajie Meng** is a lecturer at Wuhan Textile University. Her research interests include bioinformatics and data mining.

**Yi Wang** is a graduate student at Wuhan Textile University. His research interests include bioinformatics and deep learning.

**Junlin Xu** is an associate researcher at Hunan University. His research interests include bioinformatics, single cell and computational biology.

**Changcheng Lu** is a doctoral student at Hunan University. His research is bioinformatics and deep learning.

**Xianfang Tang** is a lecturer at Wuhan Textile University. His research interests include bioinformatics and data mining.

**Tao Peng** is a professor at Wuhan Textile University. His research interests include deep learning and data mining.

**Bengong Zhang** is a professor at Wuhan Textile University. His research interests include bioinformatics, single cell, data mining and computational biology.

**Geng Tian** is the president of Geneis (Beijing) Co., Ltd., Beijing, China. His research interests include genetics, bioinformatics and gene testing.

**Jialiang Yang** holds the position of Vice President at Geneis (Beijing) Co., Ltd. and serves as an adjunct professor at Changsha Medical University. His primary research interests encompass artificial intelligence, bioinformatics, medical informatics and gene testing.

## References

[1] Rifaioglu AS, Atas H, Martin MJ, et al. Recent applications of deep learning and machine intelligence on in silico drug discovery: methods, tools and databases. Brief Bioinform 2019;20:1878–912. 30084866 10.1093/bib/bby061 PMC6917215

[2] Cai L, Lu C, Xu J, et al. Drug repositioning based on the heterogeneous information fusion graph convolutional network. Brief Bioinform 2021;22:bbab319. 34378011 10.1093/bib/bbab319

[3] Ciociola AA, Cohen LB, Kulkarni P, et al. How drugs are developed and approved by the FDA: current process and future directions. Am J Gastroenterol 2014;109:620–3. 24796999 10.1038/ajg.2013.407

[4] Krantz A . Diversification of the drug discovery process. Nat Biotechnol 1998;16:1294–4. 9853592 10.1038/4243

[5] Avorn J . The $2.6 billion pill—methodologic and policy considerations. N Engl J Med 2015;372:1877–9. 25970049 10.1056/NEJMp1500848

[6] Ezzat A, Wu M, Li X-L, Kwoh CK. Computational prediction of drug–target interactions using chemogenomic approaches: an empirical survey. Brief Bioinform 2019;20:1337–57. 29377981 10.1093/bib/bby002

[7] Xu J, Zhu W, Cai L, et al. LRMCMDA: predicting miRNA-disease association by integrating low-rank matrix completion with miRNA and disease similarity information. IEEE Access 2020;8:80728–38.

[8] Meng Y, Jin M, Tang X, Xu J. Drug repositioning based on similarity constrained probabilistic matrix factorization: COVID-19 as a case study. Appl Soft Comput 2021;103:107135. 33519322 10.1016/j.asoc.2021.107135 PMC7825831

[9] Tang X, Cai L, Meng Y, et al. Indicator regularized non-negative matrix factorization method-based drug repurposing for COVID-19. Front Immunol 2021;11:603615. 33584672 10.3389/fimmu.2020.603615 PMC7878370

[10] Xu J, Cai L, Liao B, et al. CMF-Impute: an accurate imputation tool for single-cell RNA-seq data. Bioinformatics 2020;36:3139–47. 32073612 10.1093/bioinformatics/btaa109

[11] Ai C, Yang H, Ding Y, et al. Low rank matrix factorization algorithm based on multi-graph regularization for detecting drug-disease association. IEEE/ACM Trans Comput Biol Bioinform 2023;20:1–11. 37159322 10.1109/TCBB.2023.3274587

[12] Mongia A, Chouzenoux E, Majumdar A. Computational prediction of drug-disease association based on graph-regularized one bit matrix completion. IEEE/ACM Trans Comput Biol Bioinform 2022;19:3332–9. 35816539 10.1109/TCBB.2022.3189879

[13] Xu J, Meng Y, Peng L, et al. Computational drug repositioning using similarity constrained weight regularization matrix factorization: a case of COVID-19. J Cell Mol Med 2022;26:3772–82. 35644992 10.1111/jcmm.17412 PMC9258716

[14] Zeng X, Xiang H, Yu L, et al. Accurate prediction of molecular properties and drug targets using a self-supervised image representation learning framework. Nat Mach Intell 2022;4:1004–16.

[15] Xu J, Xu J, Meng Y, et al. Graph embedding and Gaussian mixture variational autoencoder network for end-to-end analysis of single-cell RNA sequencing data. Cell Rep Methods 2023;3:100382. 36814845 10.1016/j.crmeth.2022.100382 PMC9939381

[16] Jiang Y, Yang M, Wang S, et al. Emerging role of deep learning-based artificial intelligence in tumor pathology. Cancer Commun 2020;40:154–66. 10.1002/cac2.12012 PMC7170661 32277744

[17] Craik A, He Y, Contreras-Vidal JL. Deep learning for electroencephalogram (EEG) classification tasks: a review. J Neural Eng 2019;16:031001. 30808014 10.1088/1741-2552/ab0ab5

[18] Zeng X, Zhu S, Liu X, et al. deepDR: a network-based deep learning approach to in silico drug repositioning. Bioinformatics 2019;35:5191–8. 31116390 10.1093/bioinformatics/btz418 PMC6954645

[19] Yu Z, Huang F, Zhao X, et al. Predicting drug–disease associations through layer attention graph convolutional network. Brief Bioinform 2021;22:bbaa243. 33078832 10.1093/bib/bbaa243

[20] Sun X, Wang B, Zhang J, Li M. Partner-specific drug repositioning approach based on graph convolutional network. IEEE J Biomed Health Inform 2022;26:5757–65. 35921345 10.1109/JBHI.2022.3194891

[21] Li Z, Huang Q, Chen X, et al. Identification of drug-disease associations using information of molecular structures and clinical symptoms via deep convolutional neural network. Front Chem 2020;7:924. 31998700 10.3389/fchem.2019.00924 PMC6966717

[22] Zhao B-W, Hu L, You Z-H, et al. HINGRL: predicting drug–disease associations with graph representation learning on heterogeneous information networks. Brief Bioinform 2022;23:bbab515. 34891172 10.1093/bib/bbab515

[23] Meng Y, Lu C, Jin M, et al. A weighted bilinear neural collaborative filtering approach for drug repositioning. Brief Bioinform 2022;23:bbab581. 35039838 10.1093/bib/bbab581

[24] Luo H, Li M, Wang S, et al. Computational drug repositioning using low-rank matrix approximation and randomized algorithms. Bioinformatics 2018;34:1904–12. 29365057 10.1093/bioinformatics/bty013

[25] Luo H, Li M, Yang M, et al. Biomedical data and computational models for drug repositioning: a comprehensive review. Brief Bioinform 2021;22:1604–19. 32043521 10.1093/bib/bbz176

[26] Gottlieb A, Stein GY, Ruppin E, Sharan R. PREDICT: a method for inferring novel drug indications with application to personalized medicine. Mol Syst Biol 2011;7:496. 21654673 10.1038/msb.2011.26 PMC3159979

[27] Wishart DS, Knox C, Guo AC, et al. DrugBank: a comprehensive resource for in silico drug discovery and exploration. Nucleic Acids Res 2006;34:D668–72. 16381955 10.1093/nar/gkj067 PMC1347430

[28] Hamosh A, Scott AF, Amberger JS, et al. Online Mendelian Inheritance in Man (OMIM), a knowledgebase of human genes and genetic disorders. Nucleic Acids Res 2005;33:D514–7. 15608251 10.1093/nar/gki033 PMC539987

[29] Luo H, Wang J, Li M, et al. Drug repositioning based on comprehensive similarity measures and bi-random walk algorithm. Bioinformatics 2016;32:2664–71. 27153662 10.1093/bioinformatics/btw228

[30] Liang X, Zhang P, Yan L, et al. LRSSL: predict and interpret drug–disease associations based on data integration using sparse subspace learning. Bioinformatics 2017;33:1187–96. 28096083 10.1093/bioinformatics/btw770

[31] Veličković P, Cucurull G, Casanova A, et al. Graph attention networks. stat 1050.20 (2017):10–48550.

[32] Kinga D, Adam JB. A method for stochastic optimization. In: International Conference on Learning Representations (ICLR). 2015, Vol. 5.

[33] Smith LN . Cyclical learning rates for training neural networks. In: 2017 IEEE Winter Conference on Applications of Computer Vision (WACV). IEEE, 2017.

[34] Kang H, Hou L, Gu Y, et al. Drug–disease association prediction with literature based multi-feature fusion. Front Pharmacol 2023;14:1205144. 37284317 10.3389/fphar.2023.1205144 PMC10239876

[35] Zhang W, Yue X, Lin W, et al. Predicting drug-disease associations by using similarity constrained matrix factorization. BMC Bioinformatics 2018;19:1–12. 29914348 10.1186/s12859-018-2220-4 PMC6006580

[36] Wang Y, Deng G, Zeng N, et al. Drug-disease association prediction based on neighborhood information aggregation in neural networks. IEEE Access 2019;7:50581–7.

[37] Cao E, Wang D, Huang J, et al. Open knowledge enrichment for long-tail entities. In: Proceedings of The Web Conference 2020;2020, pp. 384–94.

[38] Aarsland D, Hutchinson M, Larsen J. Cognitive, psychiatric and motor response to galantamine in Parkinson's disease with dementia. Int J Geriatr Psychiatry 2003;18:937–41. 14533126 10.1002/gps.949

[39] Ikebe S-I, Harada T, Hashimoto T, et al. Prevention and treatment of malignant syndrome in Parkinson's disease: a consensus statement of the malignant syndrome research group. Parkinsonism Relat Disord 2003;9:47–9. 10.1016/s1353-8020(02)00123-2 12735915

[40] Gómez-Esteban JC, Zarranz JJ, Velasco F, et al. Use of ziprasidone in parkinsonian patients with psychosis. Clin Neuropharmacol 2005;28:111–4. 15965308 10.1097/01.wnf.0000164297.91643.ff

[41] Lao J, Madani J, Puértolas T, et al. Liposomal doxorubicin in the treatment of breast cancer patients: a review. J Drug Deliv 2013;2013:1–12. 10.1155/2013/456409 PMC3619536 23634302

[42] Li J, Chen W, Zhang P, Li N. Topoisomerase II trapping agent teniposide induces apoptosis and G2/M or S phase arrest of oral squamous cell carcinoma. World J Surg Oncol 2006;4:1–7. 16822322 10.1186/1477-7819-4-41 PMC1543631

[43] Schultze E, Buss J, Coradini K, et al. Tretinoin-loaded lipid-core nanocapsules overcome the triple-negative breast cancer cell resistance to tretinoin and show synergistic effect on cytotoxicity induced by doxorubicin and 5-fluororacil. Biomed Pharmacother 2017;96:404–9. 29031198 10.1016/j.biopha.2017.10.020

[44] Trott O, Olson AJ. AutoDock Vina: improving the speed and accuracy of docking with a new scoring function, efficient optimization, and multithreading. J Comput Chem 2010;31:455–61. 19499576 10.1002/jcc.21334 PMC3041641

[45] Colman I, Rothney A, Wright S, et al. Use of narcotic analgesics in the emergency department treatment of migraine headache. Neurology 2004;62:1695–700. 15159464 10.1212/01.wnl.0000127304.91605.ba

