# Supplementary material for: Drug repositioning based on weighted local information augmented graph neural network
Source: Brief Bioinform. 2023 Nov 28;25(1):bbad431. doi: 10.1093/bib/bbad431 (PMC10686358; doi:10.1093/bib/bbad431)
Supplement: supplementary_bbad431 [file supplementary_bbad431.docx]

**Drug repositioning based on weighted local information augmented graph neural network**

**Supplementary Figure S1.** All methods' corresponding ROC and PR curves in 10-fold-cross-validation of Fdataset, Cdataset, and LRSSL datasets, respectively.


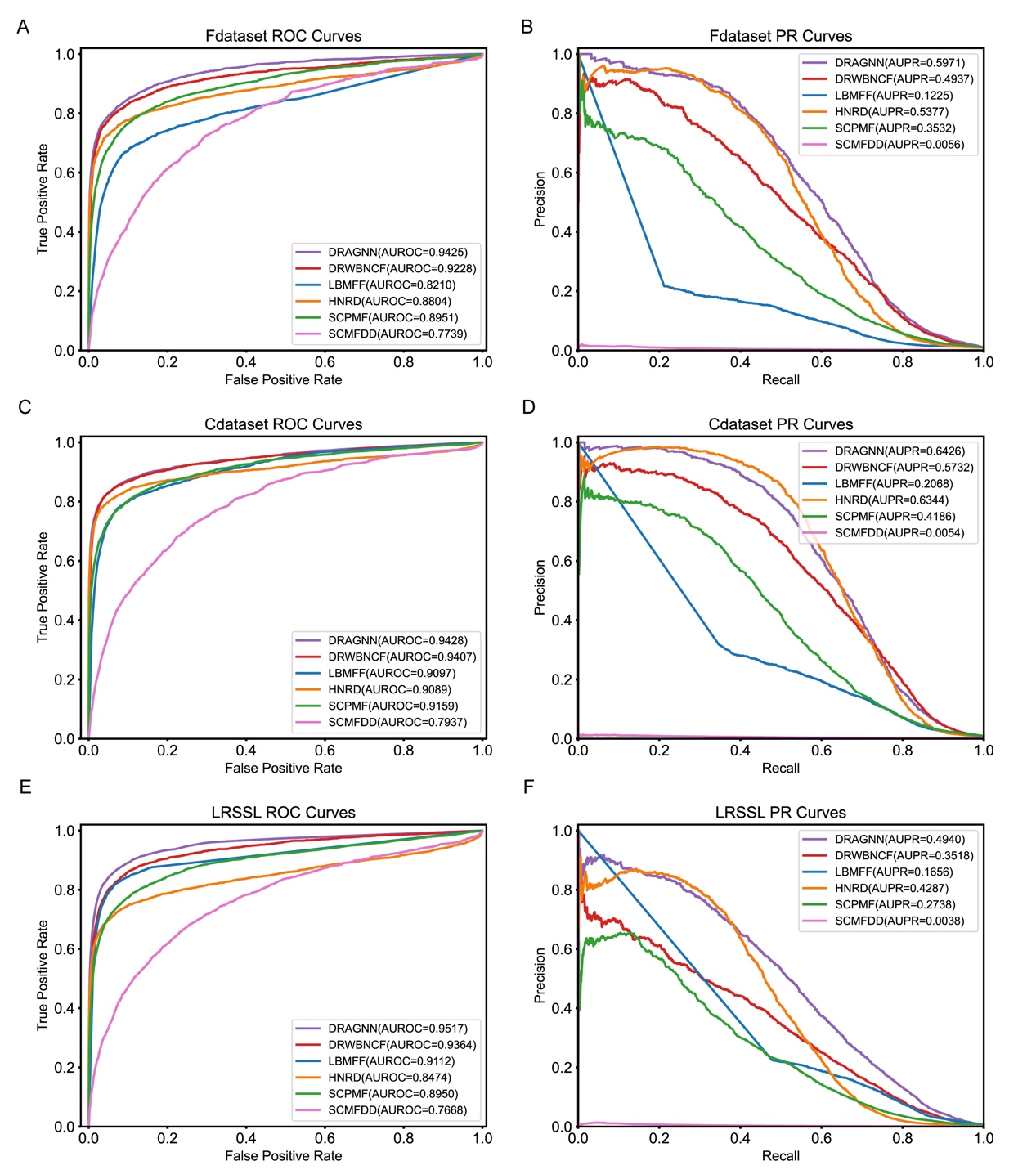


**Supplementary Table S1**. AUROCs and AUPRs of all methods in predicting candidates for new diseases on Cdataset

| Methods | AUROC | AUPR |
| --- | --- | --- |
| DRAGNN  DRWBNCF  LBMFF  HNRD  SCPMF  SCMFDD | **0.77748**  0.76462  0.73432  0.62756  0.75720  0.63235 | **0.09977**  0.09383  0.02394  0.04762  0.07489  0.02002 |

The best reported result is bolded and the second best result is underlined.

**Supplementary Table S2**. The molecular binding energies between the top 10 DRAGNN-predicted candidate drugs for BC and five target proteins(kcal/mol)

| Drug | the molecular binding energies between the drugs and five target proteins（kcal/mol） | | | | |
| --- | --- | --- | --- | --- | --- |
|  | 3hb5 | 1y98 | 4ifi | 4y2g | 6wcd |
| Doxorubicin  Cisplatin  Docetaxel  Methotrexate  Vincristine  Bleomycin  Dinoprostone  Tretinoin  Teniposide  Paclitaxel | -9.6  -1.3  -15.2  -7.7  -14.0  -8.0  -6.3  -8.3  -10.3  -13.0 | -9.1  -1.5  -12.0  -7.5  -11.6  -7.1  -5.4  -7.5  -9.3  -11.3 | -9.3  -1.4  -12.6  -7.1  -11.5  -6.9  -5.8  -7.1  -10.0  -12.3 | -8.9  -1.3  -12.1  -7.3  -12.9  -8.6  -5.9  -7.7  -9.4  -13.3 | -9.5  -1.7  -14.7  -8.2  -13.7  -9.0  -6.9  -7.5  -11.4  -13.9 |
